# First-in-Human Real-Time MR-Guided Ventricular Ablation for Idiopathic Outflow Tract Premature Ventricular Complexes

**DOI:** 10.1001/jamacardio.2025.3000

**Published:** 2025-09-17

**Authors:** Marco J. W. Götte, Luuk H. G. A. Hopman, Pranav Bhagirath, Michiel J. B. Kemme, Jules L. Nelissen, Katherine Lindborg, Axel J. Krafft, Marieke E. S. Sprengers, Steven A. J. Chamuleau, Cornelis P. Allaart

**Affiliations:** 1 Department of Cardiology, Amsterdam University Medical Centers, Amsterdam, the Netherlands; 2 Department of Radiology and Nuclear Medicine, Amsterdam University Medical Centers, Amsterdam, the Netherlands; 3 Imricor Medical Systems, Burnsville, Minnesota; 4 Siemens Healthineers AG, Erlangen, Germany

## Abstract

**Importance:**

Catheter ablation is a cornerstone therapy for symptomatic ventricular arrhythmias, yet current techniques rely on fluoroscopy and electroanatomic mapping, which provide limited soft-tissue detail and expose patients and staff to ionizing radiation. Real-time magnetic resonance (MR)–guided ablation may overcome these limitations by enabling direct visualization of cardiac anatomy, substrate, and lesion formation, all within a radiation-free environment.

**Objective:**

To demonstrate the technical feasibility and safety of the first-in-human real-time MR-guided radiofrequency ventricular ablation procedure.

**Design, Setting, and Participant:**

This was a prospective, worldwide-first roll-in case from the ongoing VISABL-VT nonrandomized clinical trial assessing MR-guided radiofrequency ablation of ventricular tachycardia. The procedure and analysis were performed in April 2025 at an academic tertiary care center equipped with a standard 1.5-T MR imaging (MRI) suite and dedicated MR-compatible electrophysiology platform, MR-compatible 12-lead electrocardiographic monitoring and recording system, MR-conditional defibrillator, and real-time catheter tracking integrated with the MRI scanner for synchronized imaging and ablation. The patient was a 73-year-old man with symptomatic, drug-refractory outflow tract premature ventricular complexes (PVCs).

**Intervention:**

The ablation was performed under general anesthesia inside the MRI scanner. Intraprocedurally acquired noncontrast 3-dimensional MR angiographic imaging was used to create an anatomical roadmap for the procedure. Real-time catheter tracking and activation mapping were performed using actively tracked diagnostic and ablation catheters. Mapping identified earliest activation first in the posterior septal right ventricular outflow tract, where ablation transiently suppressed PVCs. Ectopy recurred but was ultimately resolved by ablation performed via a retrograde aortic approach in the left coronary cusp. Lesion formation was confirmed via postprocedural MRI.

**Main Outcomes and Measures:**

Suppression of PVCs and presence or absence of procedural complications.

**Results:**

The procedure was performed under real-time MRI guidance without complications. PVCs were completely suppressed, with no recurrence during 30-minute observation or at 2-month follow-up.

**Conclusions:**

This first-in-human case demonstrates that ventricular ablation can be safely and effectively performed entirely under real-time MR guidance. Further evidence from the VISABL-VT trial will clarify clinical utility and long-term outcomes.

## Key Points

**Question:**

Can real-time magnetic resonance (MR)–guided radiofrequency ablation be safely and effectively performed for ventricular arrhythmias without fluoroscopy?

**Findings:**

In this first-in-human case from the VISABL-VT trial, a 73-year-old man underwent successful MR-guided ablation for symptomatic premature ventricular complexes using an MR-compatible electrophysiology platform, with complete ectopy suppression and no complications during or after the procedure.

**Meaning:**

This case demonstrates the technical feasibility and safety of entirely real-time MR-guided ventricular ablation, supporting further clinical investigation.

## Teaser

This case report demonstrates the technical feasibility and safety of the first-in-human real-time magnetic resonance (MR)–guided radiofrequency ventricular ablation procedure for outflow tract premature ventricular complexes.

## Introduction

Catheter ablation is an established treatment for symptomatic ventricular arrhythmias.^1^ Conventional ablation workflows typically rely on fluoroscopy guidance and electroanatomic mapping (EAM) systems to navigate catheters and deliver ablation lesions. However, these approaches have important limitations: fluoroscopy provides limited soft-tissue contrast and exposes both patients and operators to ionizing radiation, while EAM systems provide the arrhythmogenic substrate and myocardial scar only indirectly through voltage mapping or endocardial conduction properties.^2^

Magnetic resonance (MR) imaging (MRI) offers substantial promise as an advanced procedural imaging modality for guiding electrophysiological procedures. In recent years, selected atrial arrhythmia ablation cases have been successfully performed with the patient positioned inside the scanner bore,^3,4,5^ an advancement that required significant adaptations to the interventional environment, including the development of MR-compatible catheters, real-time imaging and catheter tracking, navigation software, and fully integrated electrophysiological platforms.^6^

These technological barriers have now largely been overcome, laying the groundwork for safe and effective MR-guided ventricular procedures. MRI offers several advantages in the setting of ventricular ablations: superior soft-tissue contrast, 2-dimensional (2D) in-plane and 3D imaging, integration of functional imaging, and a fully radiation-free workflow. In addition, cardiovascular MRI (CMR) permits assessment of myocardial tissue, supporting both the evaluation of acute lesion formation and the monitoring of postprocedural tissue remodeling.^7^

Despite these advantages, real-time MR guidance has not yet been used to perform ventricular ablation in humans. To our knowledge, this report describes the first-in-human case of ventricular ablation performed entirely under real-time MR guidance, without the use of fluoroscopy.

## Methods

**Figure 1.**
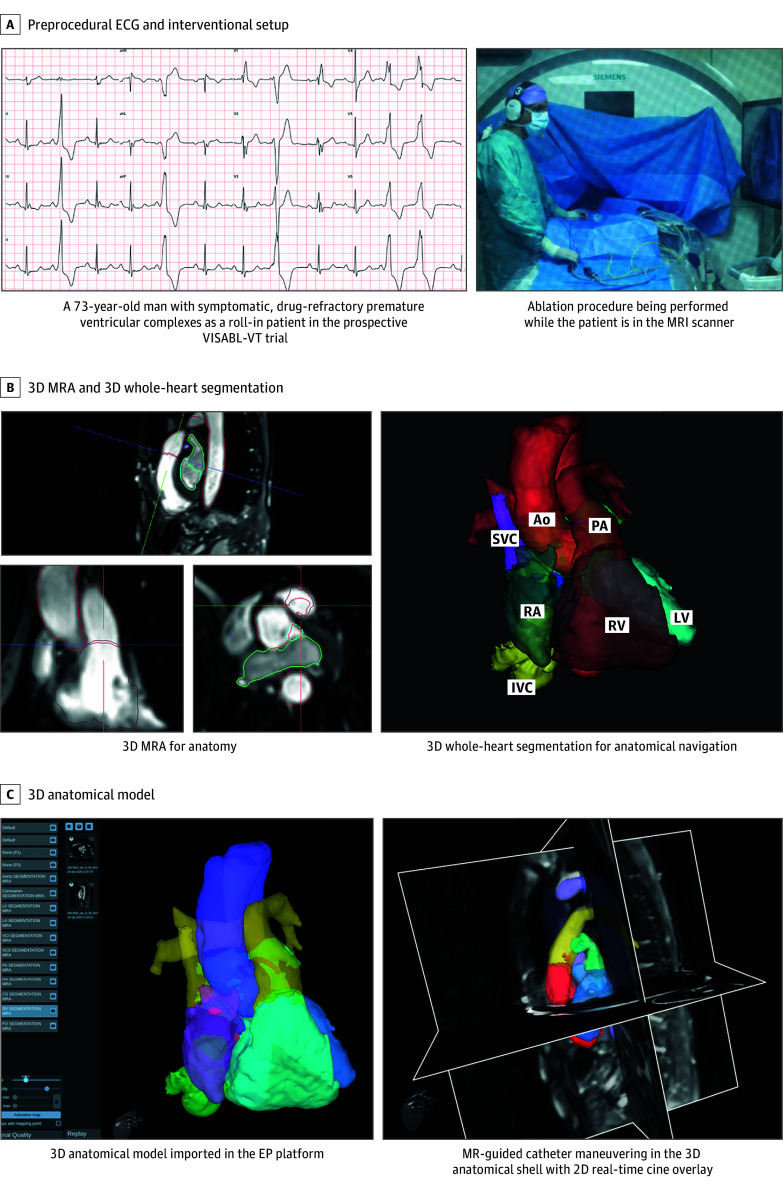
Setup and Procedural Workflow for Magnetic Resonance (MR)–Guided Premature Ventricular Complex Ablation Using an MR-Compatible Electrophysiological (EP) Platform A, Twelve-lead electrocardiogram (ECG) obtained prior to the procedure, demonstrating frequent premature ventricular complexes, and interventional setup showing the operator manipulating and navigating the catheter using real-time imaging displays while the patient is positioned inside the MR imaging (MRI) scanner bore. B, Multiplanar reconstruction of the 3-dimensional (3D) MR angiography (MRA) dataset, providing detailed anatomical visualization, and anatomical segmentation generated from the 3D MRA, highlighting cardiac structures relevant for catheter guidance. C, The segmented 3D anatomical model imported into the Northstar EP navigation system (Imricor Medical Systems), and real-time 2D cine MRI overlaid on the 3D anatomical map to facilitate catheter localization. Ao indicates aorta; IVC, inferior vena cava; LV, left ventricle; RA, right atrium; RV, left ventricle; SVC, superior vena cava.

A 73-year-old man with symptomatic outflow tract premature ventricular complexes (PVCs), refractory to antiarrhythmic therapy, was included as a roll-in patient in the prospective VISABL-VT nonrandomized clinical trial (ClinicalTrials.gov identifier: NCT05543798), designed to evaluate the safety and efficacy of MR-guided radiofrequency ablation of ventricular tachycardia (Figure 1A). The study was approved by the institutional review board, and written informed consent was obtained from the patient. The procedure and analysis were performed in April 2025.

The procedure was performed under general anesthesia in a conventional MRI suite using a 1.5-T scanner (MAGNETOM Avanto fit on VE11E; Siemens Healthineers), in conjunction with the electrophysiological platform (NorthStar and Advantage-MR; Imricor Medical Systems), MR-compatible 12-lead electrocardiographic (ECG) recording system (Mirtle Medical), and MR-conditional external defibrillator (MIPM Mammendorfer Institut für Physik und Medizin GmbH) (Figure 1A). The electrophysiological platform incorporated an actively tracked diagnostic and ablation catheter (Vision MR catheter; Imricor Medical Systems) and interfaced with the MRI scanner and 12-lead ECG setup, enabling synchronized control of imaging, catheter tracking, and EAM.^8^

Vascular access was obtained via the right femoral vein outside the MRI scanner, in the preparation room. The patient was then transferred into the MRI suite, where catheter flushing and final preparations were completed. The catheters were subsequently inserted, with MR visualization commencing approximately halfway along the inferior vena cava, when the catheters entered the MR field of view. From that point onward, catheter advancement and manipulation were performed under continuous real-time MR guidance. One catheter was positioned in the coronary sinus, while the second was used for activation mapping and subsequent ablation.

Both preprocedural anatomical imaging and postablation lesion assessment were performed within the same interventional session. A high-resolution, noncontrast MR angiogram was used to generate a 3D whole-heart anatomical shell via dedicated segmentation software (ADAS 3D; ADAS Medical) (Figure 1B). This anatomical shell was then imported into the electrophysiological platform to serve as a spatial reference and guide procedural navigation (Figure 1C).

During the procedure, only ectopic beats with consistent 12-lead morphology, coupling interval, and stability during catheter manipulation were included for mapping, while fused and catheter-induced PVCs were excluded. Ablation was performed using standard power (30-50 watts; maximum 60 seconds) and irrigation parameters for ventricular substrates, with continuous monitoring of catheter contact and stability via active tracking.

## Results

**Figure 2.**
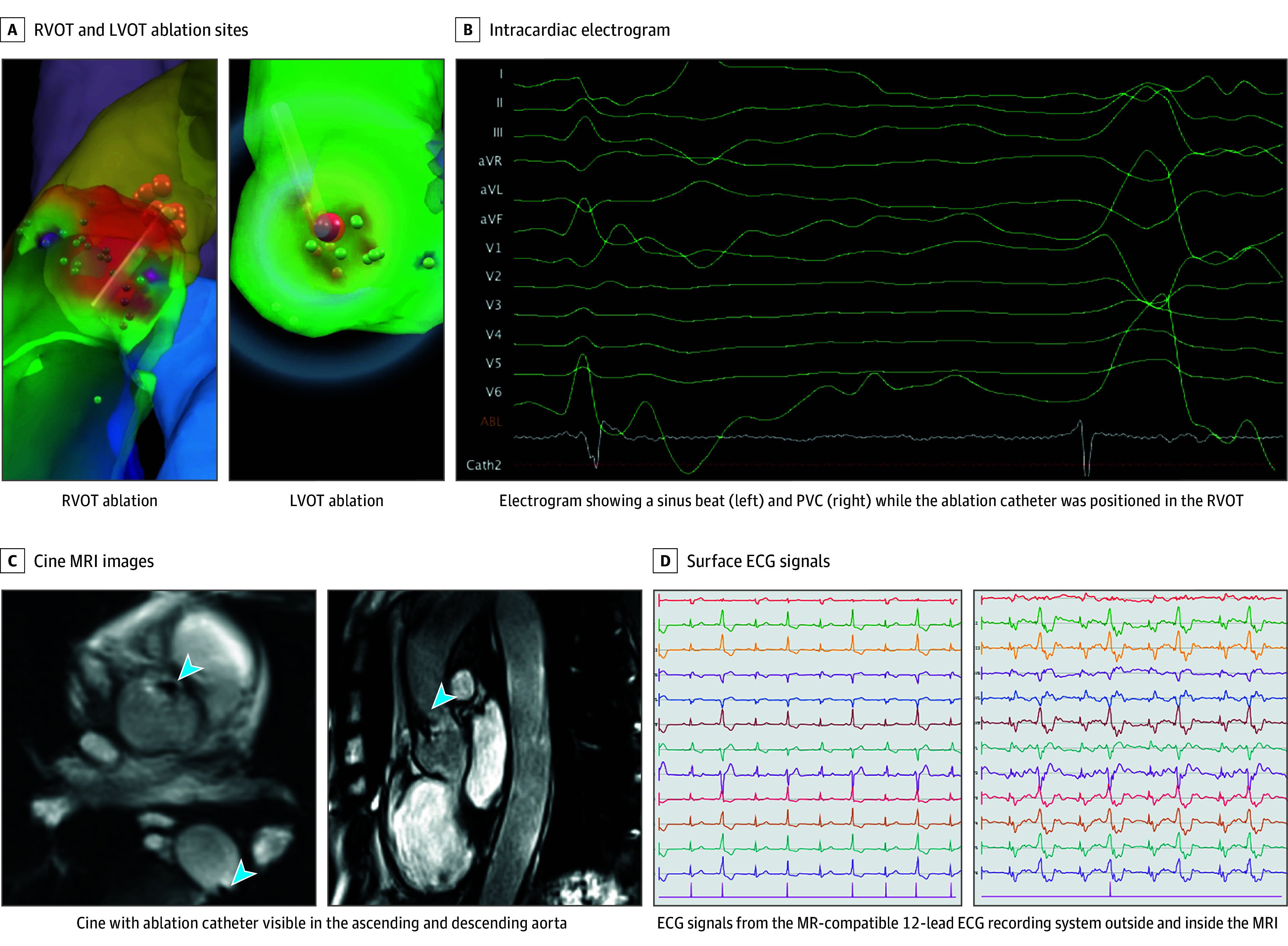
Magnetic Resonance (MR)–Guided Repositioning From Right Ventricular Outflow Tract (RVOT) to Left Ventricular Outflow Tract (LVOT) Ablation for Effective Premature Ventricular Complex (PVC) Elimination A, Visualization of the RVOT and LVOT ablation sites within the 3-dimensional anatomical shell used for catheter navigation. B, Intracardiac electrogram recorded during PVC occurrence while the ablation catheter was positioned in the RVOT, confirming electrical correspondence with the ectopic focus. C, Cine MR imaging (MRI) images showing the ablation catheter as a small susceptibility artifact (arrowheads) in both the ascending and descending aorta during retrograde access to the LVOT. D, Surface electrocardiographic (ECG) signals recorded using the MR-compatible 12-lead ECG system, demonstrating substantial artifacts due to the magnetohydrodynamic effect when the patient is inside the MRI bore (right), even in the absence of active scanning, compared with the cleaner signals recorded outside the bore (left).

Activation mapping initially localized the earliest ventricular activation at the posterior septal aspect of the right ventricular outflow tract (RVOT), where radiofrequency ablation resulted in transient suppression of PVCs (Figure 2A and B). However, the ectopy recurred shortly after ablation, and additional lesions at this site proved ineffective. Under continued real-time MR guidance, the ablation catheter was advanced retrogradely through the aorta into the left ventricular outflow tract (LVOT) (Figure 2C). Mapping revealed the earliest activation in the left coronary cusp at the junction of the left and right coronary cusp (Figure 1A). Ablation at this site led to complete suppression of spontaneous PVCs, with no recurrence during a 30-minute postablation observation period.

**Figure 3.**
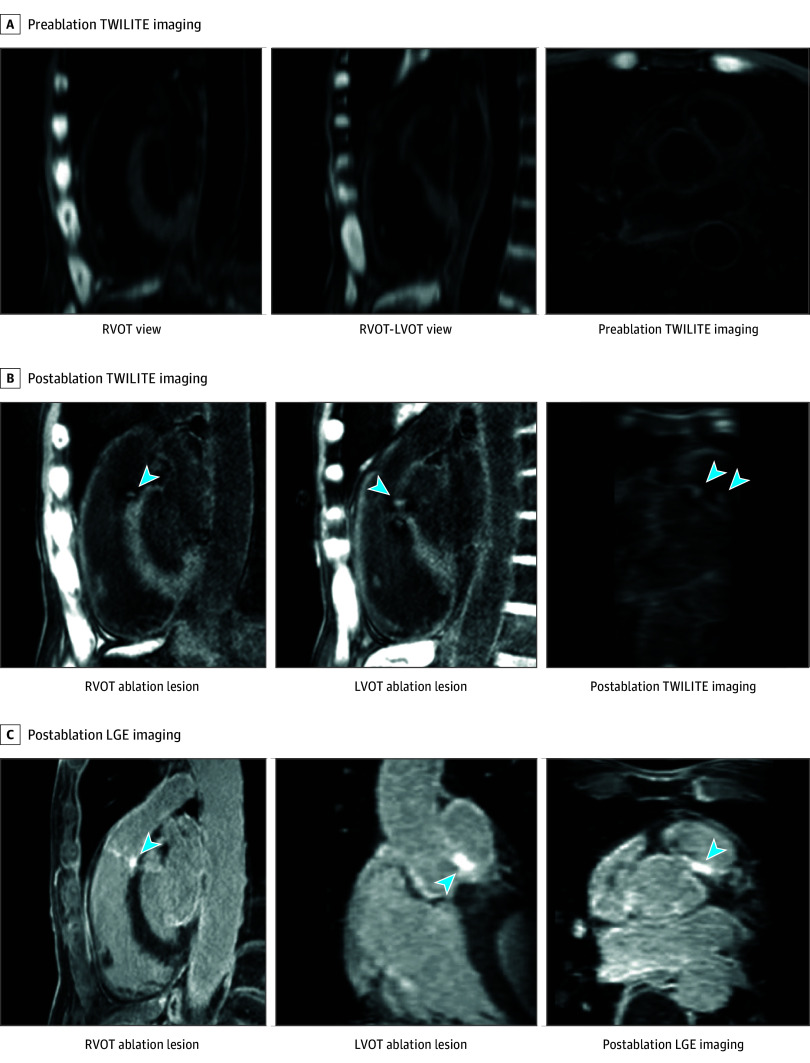
Postablation Lesion Visualization Using T1-Weighted Inversion-Recovery Prepared Long Inversion Time (TWILITE) and Late Gadolinium Enhancement (LGE) Imaging A, Preablation TWILITE imaging showing no evidence of hyperintense signal at the targeted ablation sites, confirming the absence of preexisting lesions. B, Postablation TWILITE imaging demonstrating distinct focal hyperintense lesions (arrowheads) in both the right ventricular outflow tract (RVOT) and the corresponding region of the left ventricular outflow tract (LVOT), consistent with acute ablation injury. C, Postablation LGE imaging revealing a broader area of hyperenhancement (arrowheads) encompassing both RVOT and LVOT lesions, likely reflecting a combination of ablation necrosis and surrounding tissue edema.

Postablation CMR, including 3D T1-weighted inversion-recovery prepared long inversion time (TWILITE)^9^ and late gadolinium enhancement (LGE) imaging, confirmed lesion formation at the targeted sites (Figure 3). TWILITE imaging demonstrated well-delineated lesions at both the RVOT and LVOT target areas, which were absent on the preablation TWILITE scan. Postablation LGE imaging showed enhancement in the same regions. However, the enhancement appeared more diffuse, likely reflecting gadolinium accumulation not only within the ablation core but also in the surrounding edematous tissue. Thus, TWILITE imaging appeared more specific for acute ablation injury and offers the added advantage of not requiring contrast administration.

No procedural complications were observed through 7 days of follow-up. Although Holter monitoring has not yet been performed following ablation, the patient reported complete symptom resolution during telephonic follow-up at 2 months.

## Discussion

This successful PVC ablation represents a next step toward broader implementation of interventional CMR (iCMR) procedures for arrhythmia ablation. Importantly, iCMR may be especially advantageous in cases involving complex arrhythmogenic substrates, challenging anatomical configurations, repeated ablations, or patient populations where minimizing ionizing radiation exposure is critical.^10^ Looking forward, integration of advanced imaging tools, such as real-time MR thermometry, real-time 3D updated roadmap, and quantitative lesion assessment techniques, may further enhance procedural safety and efficacy.

Several procedural and technical adaptations were necessary to enable catheter ablation within the MR environment, including real-time electrogram acquisition, reliable clinical interpretation of MR-compatible 12-lead ECGs (Figure 2D), and the availability of an MR-conditional external defibrillator. Despite these innovations, notable challenges remain. To optimize workflow, a burst-mode, interrupted real-time tracking protocol was combined with smoothing techniques, allowing for more stable ECG assessment during catheter manipulation and reducing the impact of imaging latency during rapid catheter movement. Additionally, only biphasic energy delivery is currently available within the MR-compatible setup, with no option for monophasic waveform ablation.

Furthermore, although no complications occurred in this case, structural VT ablation is associated with higher procedural risks, highlighting the need for dedicated workflows and MRI-specific safety protocols. Effective emergency management requires a well-trained team with clearly defined roles. Prior to this PVC ablation, a complete rehearsal of the bailout procedure, including rapid patient evacuation from the scanner, safe transport out of the MRI zone, and defibrillation readiness, was performed to ensure team preparedness. Repeated training is essential to minimize the time spent evacuating the MRI environment. Notably, the MR-conditional external defibrillator used in this research setting is not yet certified for clinical use and can only deliver unsynchronized shocks, which may be inadequate in the event of certain VT scenarios.

### Limitations

This study has several limitations. The ECG signal quality was limited by magnetohydrodynamic effects and, occasionally, radiofrequency interference. The MR-conditional external defibrillator is not yet certified for clinical use and can only deliver unsynchronized shocks, which may be inadequate in the event of certain VT scenarios. The performance of the current system does not yet meet the standards of conventional electrophysiological systems, with evidence suggesting that the signal-to-noise ratio, in particular, remains suboptimal. This is relevant because it may limit the detection of small prepotentials at successful ablation sites if they fall within the present background noise. Moreover, we observed a slight delay between the electrogram and the surface ECG, which may have contributed to suboptimal timing. This issue has since been resolved through a software update that corrects for timing discrepancies between the electrogram and surface ECG.

## Conclusions

This initial experience establishes foundation for real-time MR-guided ventricular ablation as a viable therapeutic approach. As the field of iCMR continues to advance, dedicated workflows, optimized MR-compatible tools, and integration with multimodal electrophysiological data will be essential to expanding clinical adoption. The ongoing VISABL-VT trial will provide further insights into procedural performance and long-term outcomes.

## Notes

Supplement. Data Sharing Statement
