# Supplementary material for: First-in-Human Real-Time MR-Guided Ventricular Ablation for Idiopathic Outflow Tract Premature Ventricular Complexes
Source: JAMA Cardiol. 2025 Sep 17;10(11):1195–200. doi: 10.1001/jamacardio.2025.3000 (PMC12444643; doi:10.1001/jamacardio.2025.3000)
Supplement: Supplement. — Data Sharing Statement [file jamacardiol-e253000-s001.pdf]

Data Sharing Statement

Götte. First-in-Human Real-Time MR-Guided Ventricular Ablation for Idiopathic Outflow Tract Premature Ventricular Complexes. *JAMA Cardiol*. Published September 17, 2025. doi:10.1001/jamacardio.2025.3000

Data
Data available: No
